# *Plasmodium *infection and its risk factors in eastern Uganda

**DOI:** 10.1186/1475-2875-9-2

**Published:** 2010-01-04

**Authors:** Rachel L Pullan, Hasifa Bukirwa, Sarah G Staedke, Robert W Snow, Simon Brooker

**Affiliations:** 1 Department of Infectious and Tropical Diseases, London School of Hygiene and Tropical Medicine, UK; 2 Uganda Malaria Surveillance Project, Mulago Hospital, Kampala, Uganda; 3 Malaria Public Health and Epidemiology Group, Kenya Medical Research Institute/Wellcome Trust Research Programme, Nairobi, Kenya; 4 Centre for Tropical Medicine, Nuffield Department of Clinical Medicine, University of Oxford, Oxford, UK

## Abstract

**Background:**

Malaria is a leading cause of disease burden in Uganda, although surprisingly few contemporary, age-stratified data exist on malaria epidemiology in the country. This report presents results from a total population survey of malaria infection and intervention coverage in a rural area of eastern Uganda, with a specific focus on how risk factors differ between demographic groups in this population.

**Methods:**

In 2008, a cross-sectional survey was conducted in four contiguous villages in Mulanda, sub-county in Tororo district, eastern Uganda, to investigate the epidemiology and risk factors of *Plasmodium *species infection. All permanent residents were invited to participate, with blood smears collected from 1,844 individuals aged between six months and 88 years (representing 78% of the population). Demographic, household and socio-economic characteristics were combined with environmental data using a Geographical Information System. Hierarchical models were used to explore patterns of malaria infection and identify individual, household and environmental risk factors.

**Results:**

Overall, 709 individuals were infected with *Plasmodium*, with prevalence highest among 5-9 year olds (63.5%). Thin films from a random sample of 20% of parasite positive participants showed that 94.0% of infections were *Plasmodium falciparum *and 6.0% were *P. malariae*; no other species or mixed infections were seen. In total, 68% of households owned at least one mosquito although only 27% of school-aged children reported sleeping under a net the previous night. In multivariate analysis, infection risk was highest amongst children aged 5-9 years and remained high in older children. Risk of infection was lower for those that reported sleeping under a bed net the previous night and living more than 750 m from a rice-growing area. After accounting for clustering within compounds, there was no evidence for an association between infection prevalence and socio-economic status, and no evidence for spatial clustering.

**Conclusion:**

These findings demonstrate that mosquito net usage remains inadequate and is strongly associated with risk of malaria among school-aged children. Infection risk amongst adults is influenced by proximity to potential mosquito breeding grounds. Taken together, these findings emphasize the importance of increasing net coverage, especially among school-aged children.

## Background

In Uganda, malaria remains the leading cause of morbidity and mortality, with an estimated 12 million clinical cases treated annually in the public health system alone [1]. To address this burden, the principal intervention strategies currently implemented include the use of insecticide-treated nets (ITNs) and long-lasting insecticide nets (LLINs), prompt effective treatment with artemisinin-based combination therapy (ACT), indoor residual spraying in regions of seasonal transmission and prevention in pregnant women. As approaches to malaria control evolve there is a continuing requirement to understand the epidemiology and determinants of malaria infection risk under varying levels of parasite transmission intensity. This is best achieved through comprehensive, community-based studies. However, consistent with a focus on the epidemiology of severe malaria morbidity [2,3], most recent studies in Uganda have been hospital-based, investigating clinical malaria among young children and pregnant women [4-7]. This report presents results from a community-based study aimed at defining the demographic and epidemiological patterns of malaria parasitaemia and to determine micro-geographic and socio-economic factors that influence the risk of infection among a community in Eastern Uganda.

## Methods

### Description of the study site

The study was conducted in four villages in Mulanda sub-county, located in Tororo district, eastern Uganda in 2008. The area is characterized by dry savannah grassland interrupted by bare rocky outcrops and lower lying swamps, although natural vegetation has mostly been replaced by cultivated crops. Average daytime temperature is 27°C, with two rainy seasons (March to May and August to October); annual rainfall is 1,000-1,500 mm^2^. The sub-county was purposively chosen as having a large population, being accessible to a health centre IV and as being representative of an area of high (stable) malaria transmission [8]. Most malaria is caused by *Plasmodium falciparum*. A 1999 survey among 1-9 year olds in Nagongera, 15 km away from Mulanda, reported a *P. falciparum *prevalence of 90.6% [9]. Estimates of entomological inoculation rates suggest that individuals receive on average 562 infective *P. falciparum *bites per year [10]. The major vector species in the region has been shown to be *Anopheles gambiae *s.s., and to a lesser extent *Anopheles funestus *[10]. No vector control activities had been undertaken in the sub-country either before or during the study. Routine malaria control in the district is typically limited to the promotion of intermittent preventive treatment during pregnancy and the distribution of insecticide-treated nets through antenatal care services; however, until now these government initiatives have had limited success [11], and no additional schemes (such as community-based mosquito net distribution campaigns) were implemented in the two years prior to this investigation.

### Census, demographic survey and mapping

Between July and December 2008, a complete census and household survey of the entire sub-county was conducted by the study team in order to gather basic demographic and socio-economic information about the target population. Study personnel systematically covered the sub-county on foot to identify and enumerate all households, and invite an adult resident (18 years of age and older) from each household to participate in a brief socio-demographic survey. Using a standardized questionnaire, information was gathered on the age and gender of each resident and socio-economic characteristics including house construction, water and sanitation, and ownership of selected household assets. A resident was defined as a person who intended to sleep primarily at that location for the subsequent six months. In total, 33,167 individuals residing in 6,397 households were enumerated in the sub-county.

Household locations were mapped using a hand-held eTrex global positioning system (GPS) receiver (Garmin Ltd., Olathe, KS). All health care facilities (public and private clinics, drugs vendors and pharmacies), open and protected water sources and other points of interest (schools, trading centres) were also geo-located using a GPS. High resolution (0.6 m) QuickBird satellite data were obtained (dated October 16, 2003) and used for geo-referencing the location of roads, infrastructure and potential mosquito breeding sites. Geographic data were compiled and maps created using ArcGIS 9.2 (Environmental Systems Research Institute Inc., Redlands, CA, USA). Nearest straight-line distances between residents' compounds and the health centre and potential mosquito breeding sites were calculated in ArcGIS.

### Recruitment and enrolment

**Figure 1:**
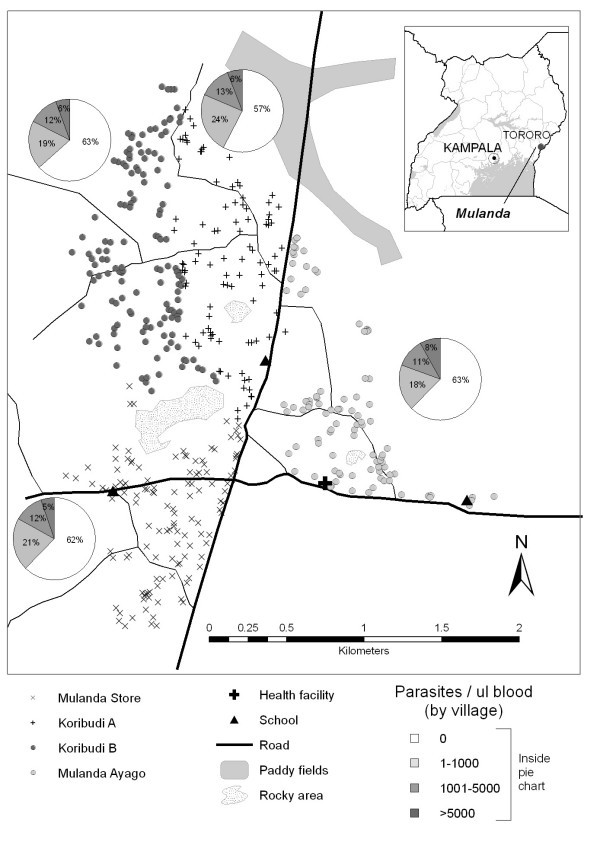
**Map of the four study villages in Mulanda sub-county, showing road networks, main geographical features and important infrastructure**. Included pie charts show the spatial distribution of prevalence and density of *Plasmodium *infection for all participating residents (n = 1844) stratified by village. *Inset: *location of the study site.

For inclusion in the present study (a total population biomedical survey describing demographic and epidemiological patterns of malaria parasitaemia within the sub-county) four villages covering an area of 7.5 km^2 ^surrounding the health centre IV were selected as geographically and socio-economically representative of the sub-county, with easy accessibility and moderate population density (Figure 1). No population weighting was used in the sampling. From September to December 2008 - which corresponds to the (later than expected) rainy season - home visitors approached all enumerated households in the four study villages to provide a brief description of the study, and to invite residents to attend an appointment at a mobile health post established for the study. Residents not at home or failing to attend their appointment were revisited up to three times over the next two weeks to assess interest in the study. Upon attending the health post, only those residents that could be unambiguously tied to a single household, resident in the study site over the last 24 months, did not work full time or attend school outside the sub-county, and were willing to give informed consent were enrolled into the study. However, excluded individuals were still offered parasitological examinations and treatment, but were not considered part of the data set for analysis.

### Study procedures

Upon enrolment a questionnaire was administered to adults and to mothers (or carers) of children to record demographic and socio-economic characteristics, medical history and mosquito net use. A clinical examination was then performed that included axillary temperature (measured using a digital thermometer) and height and weight. A rapid diagnostic test for malaria (OptiMAL; DiaMed, Cressier, Switzerland) was performed on all participants with fever (temperature > 37.2°C) or reported history of fever in the previous 24 hours. Those with a positive test but no evidence of severe illness were diagnosed with uncomplicated malaria and treated with a 6-dose regimen of co-artemether (Coartem, Novartis, 20 mg artemether/120 mg lumefantrine) in accordance with national guidelines. Research participants found by the study nurses to be suffering from severe malaria or an illness not covered by the protocol were referred to the local health facility (a county-level referral facility) for diagnosis and management as recommended by national guidelines. Finger-prick (or heal-prick from infants) blood samples were obtained to assess haemoglobin using a portable photometer (Hemocue, Angelholm, Sweden) and to prepare thin and thick blood films. Individuals were also asked to provide stool samples which were examined microscopically for the eggs of intestinal nematodes and *Schistosoma mansoni*, using the Kato-Katz technique.

Blood slides prepared for malaria were declared negative only after examination of 100 high-powered fields by two independent microscopists. Thin films from a sub-sample of 20% of parasite positive participants were read for species identification showing that 94.0% were *P. falciparum *and 6.0% were *P. malariae*; no other species or mixed infections were seen.

### Statistical analysis

All data were double-entered and cross-checked using Access (Microsoft). Statistical analyses were carried out using Stata XI software (Stata Corporation, College Station, Texas) and WinBUGs version 1.4.1 (Imperial College and Medical Research Council, London, UK). Two outcome variables were initially analysed: i) the prevalence of *Plasmodium *infection, and ii) *Plasmodium *parasite density in positive participants. However, analysis of parasite density revealed no additional insights and therefore the focus of the present analysis is on infection prevalence.

Information on ownership of household assets was used to construct a wealth index for each household using principal component analysis (PCA), using the method of Filmer and Pritchett [12]. Variables entered into the PCA included ownership of mobile phones, radios, factory-made mattresses, electric irons, motor bikes, bicycles and cattle. The first principle component explained 23.4% of the overall variability in the included variables and gave greatest weight respectively to ownership of a mobile phone (0.37), followed by radio (0.36), factory-made mattress (0.35) and watch or clock (0.35); the lowest weight was given to ownership of none of the listed items (-0.39). The resulting score was divided into quintiles, to provide a categorical measure of relative socio-economic status. Household factors potentially directly associated with infection outcomes, including toilet facilities and household construction materials, were not included in the wealth index to allow for independent assessment of their contribution.

Pearson's chi-squared test and Fisher's test were used to compare proportions between groups as appropriate. Potential risk factors for prevalence were initially investigated using frequentist logistic regression models. Explanatory variables significant at the 10% significance level were subsequently entered into a Bayesian multivariate logistic regression model, using a process of backwards-stepwise elimination. Between-household variation was taken into account by introducing into the model household-level random effects with an exchangeable correlation structure. Analysis employed a Bayesian Monte Carlo Markov Chain (MCMC) approach, which readily allows the development of random effects models [13]. Inference was based on the better fitting model, which was selected using the deviance information criteria (DIC) as a goodness of fit measure. Analysis was stratified into three age groups in order to investigate risk factors by demographic groups: pre-school children (< 5 years), school-aged children (5-15 years) and adults (≥ 16 years).

In order to assess global spatial heterogeneity in infection patterns across the whole study site, semi-variograms were generated using the R module *GeoR*. Monte Carlo envelopes (computed from random permutations of the residuals from random permutations of the data holding the corresponding locations fixed) were used to formally assess whether the data was compatible with spatial structure, under the assumption of no correlation [14,15]. Semi-variograms were also computed for households aggregated to the closest 100 m, 150 m and 200 m to overcome the problem of a small denominator in households with few residents. Variography was repeated for the random effects of the fitted Bayesian multivariate models to order assess residual spatial correlation once explanatory variables had been taken into account.

### Ethics

The study protocol was approved by the Makerere University Faculty of Medicine Research and Ethics Committee (#2008-043) Uganda National Council of Science and Technology (#HS 476) and London School of Hygiene and Tropical Medicine Ethics Committee (#5261). Prior to the start of the study, investigators met with elected government representatives and community leaders to inform them of the study and explain the methodology. Verbal consent was obtained for the brief census survey described above. Written informed consent was obtained from all adults and from parents or guardians of minors for participation in the biomedical survey; written assent was also obtained for children aged 13-18 years.

## Results

Of the 2,474 individuals from 491 households enumerated in census, 116 were no longer living in residence by time of the biomedical survey. A total of 2,358 residents living in 473 households were invited to attend the health post; 2,037 (86.4%) individuals chose to attend and could be unambiguously tied to a single household. Of these, 34 individuals were excluded from enrolment (16 because they had not been resident in the study area for the previous 24 months, and 18 because they were aged <6 months), 58 refused to provide a finger prick, 83 did not provide household socio-economic data and 19 did not provide information on bed net usage. Complete questionnaire and biomedical data was, therefore, available for 1,844 individuals from 438 households (78.2% of the total population). Individual and household characteristics for participants enrolled in the study are summarized in Table 1: comparison of census data for the individuals included in the final sample with those who chose not to participate indicates that the final study population under-sampled adult males (p < 0.001). There were however no statistical difference in the relative socio-economic status of participant and non-participant households, nor of bed net ownership (Table 1).

**Table 1:** Comparison of census population for the study villages with that for entire sub-county, and enrolled households/participants with those that chose not to participate (refusals).

Household Characteristics ^a^	Study villages	Sub-county	*P *^d^	Participants	Refusals	*P *^e^
	(n = 496)	(n = 6,397)		(n = 438)	(n = 58)	
Residents: median(range)	5 (1-15)	5 (1-15)	0.99	5 (1-10)	3 (1-14)	<0.001
Construction of home:						
Mud and wattle	78.2%	72.8%		78.8%	71.0%	
Bricks	12.2%	16.5%	0.0	11.9%	19.4%	0.25
Cement/concrete	9.6%	10.7%		9.4%	9.7%	
Own ≥ one bed net	68.2%	64.7%	0.1	67.8%	62.9%	0.4
≥ 1 net per 2 residents	20.3%	16.3%	0.1	18.1%	33.9%	0.005
Own ≥ asset ^b^	87.9%	89.1%	0.4	87.7%	87.1%	0.9
Socioeconomic group ^c^						
Poorest	20.9%	22.7%		20.1%	20.3%	
Poor	21.8%	19.6%		22.6%	22.0%	
Median	18.7%	15.0%	0.0	18.5%	11.7%	0.7
Less poor	19.3%	18.4%		19.2%	20.3%	
Least poor	19.3%	24.3%		19.6%	25.4%	

**Individual Characteristics ^a^**	**Study villages** (n = 2358)	**Sub-county** (n = 33,167)	***P *^d^**	**Participants** (n = 1844)	**Refusals** (n = 514)	***P *^e^**

Mean age in years (SD)	21.8 (21.1)	20.2 (20.0)	<0.001	21.3 (21.0)	22.6 (20.4)	<0.001
Age group						
Under 5 yrs	19.2%	19.6%		21.5%	16.1%	
School aged (5-15)	34.1%	36.0%	0.06	34.9%	29.4%	<0.001
Adults (16 +)	46.8%	44.4%		43.6%	54.6%	
Gender (% male)	49.2%	48.3%	0.3	45.2%	57.2%	<0.001

^a ^Ascertained during household census

^b ^assets include: iron, clock, factory-made mattress, bicycle, radio, television sofa, fridge, mobile phone, motorcycle and car

^c ^groups defined by scores from the first component of PCA (asset ownership) performed on census data from the four study villages

^d ^P-value: study village (all residents/households) vs. sub-county (all residents/households)

^e ^P-value: study participants vs. those resident in the study villages who chose not to participate

**Figure 2:**
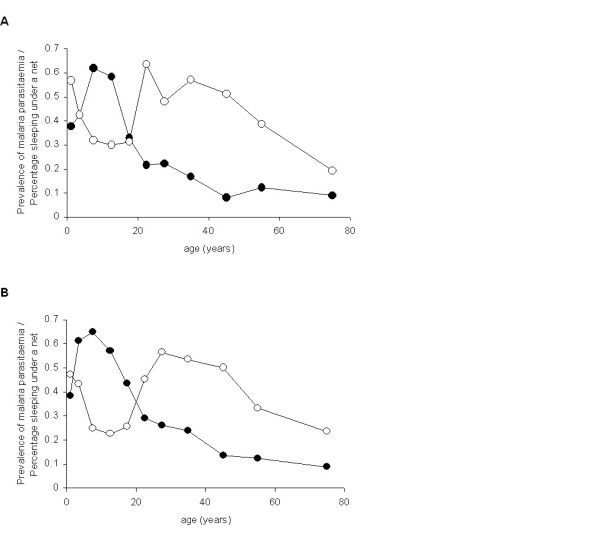
**Age patterns in the prevalence malaria parasitaemia (solid circles) and percentage reporting sleeping under a bed net the previous night (open circles) for (A) females and (B) males**.

Overall, 38.5% of individuals were infected with *Plasmodium *spp. Figure 1 shows the household distribution of presence and density of *Plasmodium *spp. parasitaemia stratified by village. Prevalence did not significantly vary between villages (χ^2 ^3.84; p = 0.3): 37.8% in Mulanda Store, 36.6% in Koribudi B, 37.8% in Mulanda Ayago and 42.6% in Koribudi A. Examination of semi-variograms for household prevalence found no evidence of consistent global spatial structure, indicating that on average across the whole study site infection was randomly distributed (data not shown). Prevalence was significantly higher among males than females (43.3% versus 34.5%, p < 0.001) and peaked among individuals aged five to nine years (63.5%), falling to 29.1% by 16 years and 12.9% in those over 30 years old (Figure 2).

The majority (67.9%) of households owned at least one mosquito net; 45.1% of which were long lasting insecticide nets (LLIN) or had been treated with an insecticide (ITN). Net ownership increased with socio-economic status, ranging from 43.2% any net/17.2% ever-treated net among households of the poorest quintile to 95.0% any net/50.0% ever-treated net among the richest quintile (χ 2, p < 0.001). However, only 9.9% of households owned at least one net per two residents, the Uganda national recommended minimum net coverage, and only 39% of participants reported having slept under a bed net the previous night. In terms of differences by age, net use was initially highest among pre-school children, sharply declining among school-aged children, before rising again across the ages 20-44 years and finally decreasing gradually in older ages; this pattern was similar among females and males (Figure 2). Reported usage in school-aged children was also strongly associated with education level of the household head and of the primary carer (both significant correlates of socio-economic status): for example, usage ranged from 17.4% among those whose primary carer had no formal education to 55.1% in those whose primary carer had attended secondary school (p < 0.001).

Results of the univariate analysis of risk factors for *Plasmodium *infection are provided in Additional File 1, both overall and stratified by age group. Overall, sex and age were identified as significant risk factors, although sex was not significantly associated after stratifying by age group. Infection risk was significantly lower among pre-school children living in households in possession of an ever-treated net and with minimum adequate mosquito net coverage, and those reported to have slept under a mosquito net the previous night. Reported net usage was associated with decreased risk among school-aged children, but not among adults. Across all age groups, living >750 m from rice growing areas and open springs (both potential mosquito breeding areas) was associated with decreased risk, whilst living >1,000 m from the health centre was associated with increased risk. Notably, relative socio-economic status was not associated with infection risk among any age group.

The results of the Bayesian multivariate random-effect logistic regression are shown in Table 2. The risk of infection was significantly reduced for those individuals reporting sleeping under a net the previous night and living >750 m from rice fields. When stratified by age group, infection risk was significantly decreased among pre-school children living in a household in possession of an ever-treated net or with minimum adequate net coverage, and among school-aged children who reported sleeping under a net the previous night. Risk in adults was defined by location of their household of residence: reduced risk among those living >750 m from rice fields and increased risk among those living >1,000 m from the health centre. Comparison of DIC values revealed that clustering within compounds appreciably improved the fit of all models, suggesting that after adjusting for bed net usage and location of the household, there was strong statistical evidence of household clustering of infection; this clustering was independent of the spatial location of households.

**Table 2:** Bayesian multivariate analysis of the prevalence of *Plasmodium *spp. parasitaemia, overall and stratified by age group

	All ages		Pre-school		School-aged		Adults	
						
	(0-85 years)		(<5 years)		(5-15 years)		(≥ 16 years)	
				
Parameter:	OR	(95% BCI)	OR	(95% BCI)	OR	(95% BCI)	OR	(95% BCI)
***Personal characteristics:***								
Age (in years):								
<2	1		1		-		-	
3-4	1.78	(1.16-2.74)	2.06	(1.22-3.56)	-		-	
5-9	3.01	(2.02-4.45)	-		-		-	
10-15	2.15	(1.46-3.23)	-		-		-	
16-25	0.59	(0.19-0.45)	-		-		1	
26-49	0.29	(0.08-0.25)	-		-		0.50	(0.31-0.78)
50 +	0.14	(0.19-0.45)	-		-		0.27	(0.15-0.48)
Age-linear term	-		-		0.94	(0.89-0.99)	-	
***Household characteristics:***								
Slept under a net last night?	0.75	(0.58-0.96)	-		0.57	(0.38-0.86)	-	
≥ 1 net per 2 residents	-		0.15	(0.02-0.72)	-		-	
LLIN/ITN in household ^a^	-		0.47	(0.23-0.89)	-		-	
Rice growing area > 750 m	0.65	(0.47-0.89)	-		-		0.49	(0.28-0.82)
Health centre >1000 m	-		-		-		1.75	(1.02-3.06)
***Variance parameter:***								
Household σ^2^	0.45	(0.20-0.76)	2.02	(0.23-4.75)	0.44	(0.04-1.14)	0.51	(0.03-1.44)

Parameters left blank were omitted in the final multivariate model for this demographic model.

^a ^LLIN long-lasting insecticidal net; ITN insecticide-treated net

## Discussion

Community-based epidemiological studies serve an important public health role since they provide contemporary estimates of infection prevalence and intervention coverage, and can identify factors whose manipulation could further prevent or control malaria in endemic populations. In Uganda, there are remarkable few recent studies of malaria within communities, with previous detailed descriptions provided by Onori and Jelliffe in the 1960s [16-20] and sentinel site studies by Okello and Talisuna from 1999-2006 [9,10,21]. The results from the present study show that in eastern Uganda *P. falciparum *infection remains highly prevalent (38.5%), although prevalence in children aged 1-9 years is lower than that reported in 1999 by Talisunu *et al *for the same age group in a neighbouring population (55.0% vs. 90.6%) [9]. Mosquito net ownership remains unacceptably low, with only 31% of households owning at least one ever-treated mosquito net (ITN or LLIN). Whilst this is higher than the 2006 Uganda regional average (24%) [11], it is still considerably lower than the government Malaria Control Programme's stated aim of 85% by 2010. Nonetheless, these results show that sleeping under a net is strongly associated with a decreased risk of infection, especially among school-aged children.

Mosquito net use was found to be lowest among school-aged children, an observation also reported during individual studies in Tanzania [22-24], South Central Somalia [25], Ethiopia [26] and Nigeria [27], and in an analysis of data from 18 national household surveys [28]. Although data were not explicitly collected on where participants acquired their bed nets, such variation by age group may be the consequence of net distribution programmes that have historically focused on providing nets to young children and pregnant women during routine clinic visits or mass-catch-up immunization campaigns. The findings may additionally be explained by household sleeping patterns with school-aged children sleeping together on separate beds from young children and their mothers. Those school-aged children who did report sleeping under a net were at nearly half the risk of malaria infection compared to those that did not. In contrast, net use was not associated with infection risk among adults, who presumably remain outside until later in the evening, increasing their likelihood of being bitten. Previous estimates of the protection afforded by mosquito nets vary widely across different settings, with cross-sectional studies providing evidence of higher protective efficacy (51-63%) [25,29,30] than estimates from randomized controlled trials (13-15%) [31]. While differences in study design and transmission settings make comparisons between studies difficult, our finding that mosquito nets offer high rates of protection in school-aged children are consistent with individual cross-sectional studies in Somalia [25] and Tanzania [32].

Notable among the results was a lack of association between infection risk and socio-economic factors. This contrasts with previous studies conducted in both rural and peri-urban settings that have reported increased risk of malaria infection in children living in poor housing [33,34] and with low socio-economic status [35-37]. The observed lack of association may be a consequence of both the high transmission setting and the generally low socio-economic status of this community. In addition, findings from Kenya suggest that whilst asset data (as used in our wealth index) may effectively capture differences in socio-economic status at national scales, expenditure data (which was not collected) may be a more appropriate measure in rural settings [38]. Although our results do suggest that risk of infection was equal across all socio-economic groups, the least poor and most educated were still significantly more likely to own and use bed nets than their poorer and less well educated counterparts. This pro-rich bias in net ownership is similar to that found in studies conducted in comparable settings, including Tanzania [24,39], Kenya [40,41], Ghana [42,43] and the Gambia [44], suggesting that the inequities estimated in this study are typical of those found in low-income settings.

Infection risk was strongly associated with proximity of households to rice-growing areas, emphasizing how this habitat may provide optimal breeding and resting sites for mosquitoes. There was however little evidence of a consistent second-order spatial pattern occurring over the entire study region as shown by variography. The relationship between malaria infection and distance from potential vector breeding sites has been reported in studies in both rural [45,46] and urban communities [36,47], with entomological studies suggesting that mosquitoes tend not to disperse far from breeding sites when blood meals (i.e. humans) and aquatic habitats are in close proximity [48-50]. Importantly however, whilst attention has focused on associations between rice agro-ecosystems, mosquito diversity and malaria transmission in Kenya and Tanzania [51-54], far less is known of the impact of this habitat type on malaria transmission in Uganda. Nonetheless, our findings are consistent with entomological studies elsewhere demonstrating the suitability of rice paddies as a larval habitat for *An. gambiae*, the most likely vector in this region [52,55]; further entomological studies are however needed to confirm this finding. Consequently, our results emphasize how an understanding of transmission heterogeneity within communities can be vastly improved by knowledge of the geographic locality of breeding sites [48].

There are a number of policy implications arising from this study. First, given the protection afforded by sleeping under a mosquito net coupled with the current low usage among school-aged children, there is a clear need to promote net use amongst school-age children. Whilst targeting individual protection to vulnerable groups is an accepted priority, recent evidence does suggest that protecting all community members can yield enhanced benefits in terms of health and social equity [56]. However, observed inequalities in ownership of bed nets across socio-economic groups in Mulanda suggest that, in order to dramatically increase universal coverage, the costs associated with acquiring a net must be reduced. Whilst there is debate surrounding the most appropriate approach to achieving equitable and sustainable ITN delivery [57-62], several studies have indicated that post-intervention inequalities in net use are lower following free distribution campaigns than following social marketing interventions [40,42,43]. Current distribution strategies in Uganda are focused on young children and pregnant women, following a mixed model that includes both the commercial sector and civil society organizations. Our findings suggest that these strategies should be extended to specifically target school children. For example, provision of free or heavily subsidized LLINs through schools and encouragement of residential (boarding) schools to provide nets in dormitories, coupled with a skills-based health education, would help to ensure that children develop the knowledge, attitudes and skills necessary to reduce their malaria risk [63].

Second, the data demonstrates a strong relationship between infection risk and proximity to rice fields, emphasizing a role for localized environmental management. There has been recent renewed interest in larval control as part of integrated malaria control strategies [64], with theoretical studies suggesting that the true effectiveness of many vector-control methods may be much greater than previously thought [65]. Encouragingly, in urban settings environmental management strategies have successfully led to reductions in adult mosquito density [66] and malaria transmission and morbidity [67,68]. Whilst malaria transmission in rural environments is typically less focal, our results do support previous observations that malaria risk in these communities is greatest near mosquito breeding sites [36,47,69,70] suggesting that some form of targeted larval control may prove a helpful supplement to the use of insecticide-treated nets.

## Conclusion

Cost-effective intervention options in malaria endemic communities need to be guided by an informed understanding of the local epidemiology of infection. These results show bed net use to be strongly associated with a decreased risk of infection in this rural Ugandan community, especially among school-aged children. Despite this, the number of individuals reporting sleeping under a bed net was inadequate, with dramatic increases in coverage required to meet current national and international targets. Findings also indicate that infection risk is influenced by proximity to potential mosquito breeding grounds, highlighting potential targets for localized environmental management. Taken together, these results underline the need for a targeted approach to integrated vector-control initiatives, further emphasizing the value of spatially explicit, community-based studies when evaluating ongoing control efforts.

## Competing interests

The authors declare that they have no competing interests.

## Authors' contributions

The study was designed by RLP and SB. RLP collected the data, with support from HB, SGS and SB. RWS provided scientific advice on analysis and interpretation. RLP developed the initial draft paper and all authors read, commented on and approved the final manuscript.

## Supplementary Material

Additional file 1 **Bivariate logistic regression models for *Plasmodium *spp. infection, stratified by age group**. The data provided represent the preliminary statistical analysis of risk factors for *Plasmodium *spp. infection, for all ages and stratified by age group. Click here for file
