# Supplementary material for: Plasmodium infection and its risk factors in eastern Uganda
Source: Malar J. 2010 Jan 4;9:2. doi: 10.1186/1475-2875-9-2 (PMC2822788; doi:10.1186/1475-2875-9-2)
Supplement: Additional file 1 — Bivariate logistic regression models for Plasmodium spp. infection, stratified by age group. The data provided represent the preliminary statistical analysis of risk factors for Plasmodium spp. infection, for all ages and stratified by age group. [file 1475-2875-9-2-S1.DOC]

| **Additional Table 1 Bivariate logistic regression models for *Plasmodium* prevalence for the whole community, and stratified by age group.** | | | | | | | | | | | | | | | | |
| --- | --- | --- | --- | --- | --- | --- | --- | --- | --- | --- | --- | --- | --- | --- | --- | --- |
|  | Whole Community (all ages) | | | | Under 5 years | | | | School-aged children (5-15 years) | | | | Adults (16 + years) | | | |
| **Explanatory variable** | *N* | OR | 95% CI | *P* | *N* | OR | 95% CI | *P* | *N* | OR | 95% CI | *P* | *N* | OR | 95% CI | *P* |
| **Demographic factors**  Sex:  Female  Male | *1010*  *834* | 1  **1.46** | -  (1.21-1.76) | -  <0.001 | *207*  *190* | 1.00  1.43 | -  (0.96-2.13) | -  0.08 | *316*  *351* | 1  1.08 | -  (0.79-1.48) | -  0.6 | *494*  *310* | 1  1.29 | -  (0.90-1.67) | -  0.2 |
| Age:  <2 years  3-4 years  5-9 years  10-15 years  16-25 years  26-49 years  50 + years | *2113*  *184*  *340*  *303*  *209*  *367*  *228* | 1  **1.73**  **2.79**  **2.15**  **0.59**  **0.32**  **0.18** | -  (1.17-2.56)  (1.98-3.94)  (1.52-3.05)  (0.39-0.88)  (0.22-0.47)  (0.11-0.29) | -  0.006  <0.001  <0.001  0.01  <0.001  <0.001 | *213*  *184* | 1.00  **1.74** | -  (1.17-2.60) | -  0.007 | *340*  *303* | 1  1.27 | -  (0.93-1.75) | -  0.2 | *209*  *367*  *228* | 1  **3.27**  **1.85** | -  (1.92-5.53)  (1.11-3.07) | -  <0.001  0.02 |
| **Bednets Use**  Slept under a net last night?  No  Yes  More than 1 net / 2 ppl  No  Yes  Ever-treated nets in hhold  No  Yes | *1128*  *716*  *1698*  *140*  *1189*  *651* | 1  **0.61**  1  **0.56**  1  1.09 | -  (0.50-0.74)  -  (0.38-0.83)  -  (0.90-1.3) | -  <0.001  -  0.004  -  0.4 | *207*  *190*  *378*  *19*  *253*  *144* | 1  **0.68**  1  **0.22**  1  **0.64** | -  (0.46-1.10)  -  (0.06-0.78)  -  (0.42-0.97) | -  0.06  -  0.02  -  0.04 | *495*  *172*  *625*  *42*  *431*  *236* | 1  **0.62**  1  0.85  1  1.20 | -  (0.44-0.88)  -  (0.45-1.61)  -  (0.86-1.67) | -  0.008  -  0.6  -  0.3 | *540*  *354*  *725*  *79*  *533*  *271* | 1  0.92  1  0.73  1  1.20 | -  (0.63-1.32)  -  (0.34-1.42)  -  (0.82-1.76) | -  0.6  -  0.4  -  0.3 |
| **Household characteristics**  Relative SES (quintiles)  Poorest  Poor  Median  Less poor  Least poor | *318*  *367*  *350*  *398*  *411* | 1  0.89  1.01  1.24  0.85 | -  (0.65-1.21)  (0.74-1.38)  (0.92-1.68)  (0.70-1.28) | -  0.4  0.9  0.2  0.7 | *75*  *90*  *85*  *75*  *72* | 1  1.42  0.81  1.81  0.85 | -  (0.76-2.63)  (0.43-1.54)  (0.95-3.45)  (0.44-1.65) | -  0.3  0.5  0.07  0.6 | *104*  *107*  *118*  *155*  *159* | 1  **0.58**  0.74  0.83  0.83 | -  (0.33-1.01)  (0.43-1.28)  (0.49-1.39)  (0.50-1.40) | -  0.05  0.3  0.5  0.5 | *139*  *170*  *147*  *168*  *180* | 1  1.00  1.60  1.30  0.90 | -  (0.54-1.85)  (0.88-2.90)  (0.72-2.25)  (0.48-1.66) | -  0.99  0.1  0.4  0.7 |
| Residents (linear 1-10+) |  | **1.18** | (1.13-1.24) | <0.001 |  | 1.06 | (0.97-1.17) | 0.2 |  | 1.06 | (0.99-1.15) | 0.1 |  | 1.05 | (0.98-1.13) | 0.1 |
| Latrine  None / uncovered pit  Covered pit | *1472*  *372* | 1  0.92 | -  (0.72-1.16) | -  0.5 | *316*  *81* | 1  0.78 | -  (0.47-1.28) | -  0.3 | *511*  *132* | 1  **0.69** | -  (0.47-1.02) | -  0.07 | *645*  *152* | 1  1.35 | -  (0.88-2.08) | -  0.2 |
| **Location characteristics**  Distance from health centre  <1000m  >1000m | *525*  *1278* | 1  **1.13** | -  (1.02-1.26) | -  0.02 | *1044*  *282* | 1  1.06 | -  (0.84-1.33) | -  0.6 | *197*  *432* | 1  1.13 | -  (0.96-1.35) | -  0.1 | *224*  *564* | 1  **1.44** | -  (1.14-1.81) | -  0.002 |
| Distance - open spring / well  < 250m  > 250m | *102*  *1696* | 1  **0.58** | -  (0.37-0.88) | -  0.01 | *25*  *372* | 1  0.56 | -  (0.22-1.43) | -  0.2 | *42*  *586* | 1  **0.53** | -  (0.26-1.08) | -  0.08 | *41*  *738* | 1  1.25 | -  (0.51-3.03) | -  0.6 |
| Distance - rice paddies  <750m  >750m | *382*  *1462* | 1  **0.70** | -  (0.55-0.88) | -  0.002 | *82*  *315* | 1  0.75 | -  (0.46-0.12) | -  0.3 | *128*  *515* | 1  0.81 | -  (0.54-1.21) | -  0.3 | *172*  *632* | -  **0.49** | -  (0.33-0.73) | -  <0.001 |
| Distance - rocky area  <500m  >500m | *1026*  *818* | 1  **1.34** | -  (1.11-1.62) | -  0.002 | *215*  *182* | 1  **1.73** | -  (1.16-2.59) | -  0.007 | *357*  *286* | 1  1.21 | -  (0.88-1.66) | -  0.3 | *454*  *350* | 1  1.37 | -  (0.95-1.97) | -  0.09 |
| Village:  Mulanda Store  Koribudi A  Koribudi B  Mulanda Ayago | *519*  *392*  *563*  *370* | 1  1.00  0.89  0.88 | -  (0.74-1.34)  (0.68-1.17)  (0.64-1.20) | -  1.0  0.4  0.4 | *106*  *82*  *129*  *80* | 1  1.29  0.89  1.36 | -  (0.73-2.30)  (0.53-1.49)  (0.76-2.43) | -  0.4  0.7  0.3 | *186*  *123*  *196*  *138* | 1  1.21  0.80  0.86 | -  (0.75-1.95)  (0.53-1.21)  (0.54-1.35) | -  0.4  0.3  0.5 | *227*  *187*  *238*  *152* | 1  **1.74**  1.21  0.78 | -  (1.06-2.84)  (0.74-1.97)  (0.43-1.34) | -  0.03  0.4  0.4`` |
| Nb. Variables significant at the 10% significance level (ORs shown in bold) were subsequently entered into Bayesian multivariate logistic regression models using a process of backwards-stepwise elimination. | | | | | | | | | | | | | | | | |
